# The Urgent Need for Recommending Physical Activity for the Management of Diabetes During and Beyond COVID-19 Outbreak

**DOI:** 10.3389/fendo.2020.584642

**Published:** 2020-10-28

**Authors:** Isabela Roque Marçal, Bianca Fernandes, Ariane Aparecida Viana, Emmanuel Gomes Ciolac

**Affiliations:** Exercise and Chronic Disease Research Laboratory (ECDR), Department of Physical Education, School of Sciences, Campus Bauru (UNESP), São Paulo State University, Bauru, Brazil

**Keywords:** burden of disease, disease severity, exercise, noncommunicable chronic diseases, mortality, pandemic (COVID-19)

## Abstract

Diabetes is the second most prevalent non-communicable chronic diseases (NCDs) in patients with coronavirus disease 2019 (COVID-19) and is highly associated with increased incidence of disease severity and mortality. Individuals with diabetes and poor glycemic control have an even worse prognosis. Despite of the need/effectiveness of social distancing measures (i.e.: home confinement, quarantine and/or lockdown) during COVID-19 outbreak, preliminary findings showed an increase in negative behaviors during COVID-19 home confinement (i.e.: ~33.5% reduction in physical activity, ~28.6% (~3.10h) increase in sedentary behavior (i.e.: daily sitting, reclining and lying down time), and more unhealthy food consumption and meal pattern), which may have important clinical implications. For example, we estimated that this reduction in physical activity can increase the cases of type 2 diabetes (from ~7.2% to ~9.6%; ~11.1 million cases per year) and all-cause mortality (from ~9.4% to ~12.5%; ~1.7 million deaths per year) worldwide. Few weeks of reduction in physical activity levels result in deleterious effects on several cardiometabolic (i.e.: glycemic control, body composition, inflammatory cytokines, blood pressure, vascular function…) and functional parameters (i.e.: cardiorespiratory/muscle fitness, balance, agility…). In contrast, physical activity and exercise are important tools for preventing and treating diabetes and others NCDs. Home-based exercise programs are useful, safe and effective for the management of diabetes, and could be widely used during COVID-19 outbreak. In this context, there is an urgent need for recommending physical activity/exercise, during and beyond COVID-19 outbreak, for improving the management of diabetes, as well as to prevent the increase in global burden of COVID-19, diabetes and others NCDs.

## Key points

- Diabetes is highly associated with increased disease severity and mortality of coronavirus disease 2019 (COVID-19), and poor glycemic control have an even worse prognosis.
- Physical inactivity during home confinement may increase global cases of type 2 diabetes (more ~11.1 million cases per year) and all-cause mortality (more ~1.7 million deaths per year), and impairs several cardiometabolic (i.e.: glycemic control, body composition, inflammatory cytokines, blood pressure, vascular function…) and functional parameters (i.e.: cardiorespiratory/muscle fitness, balance, agility…).
- Physical activity and exercise are important tools for preventing and treating diabetes and others noncommunicable chronic diseases (NCDs); home-based exercise programs are a useful, safe, and effective strategy that could be widely used during COVID-19 outbreak.
- There is an urgent need for recommending physical activity/exercise, during and beyond COVID-19 outbreak, for improving the management of diabetes, as well as to prevent the increase of global burden of COVID-19, diabetes and others NCDs.

## Introduction

The pandemic of coronavirus disease 2019 (COVID‐19) is an unprecedented public health emergency of global concern that resulted in more than 813.406 deaths worldwide in only 8 months (as of August 24, 2020) (1). Individuals with non-communicable chronic diseases (NCDs) are at high-risk of severe cases and mortality for COVID-19 (2, 3). Diabetes is the second most prevalent NCDs in individuals requiring treatment for COVID-19 (prevalence = 10.0%, 95% confidence interval [CI] 8.0% to 12.0%) and is highly correlated with disease severity (odds ratio [OR] 2.61, 95% CI 1.93 to 3.52) (2). In addition, a recent study showed that COVID-19 patients with diabetes required more medical interventions, and had higher mortality (7.8% *versus* 2.7%, adjusted hazard ratio [HR] = 1.49) and multiple organ injury than those without diabetes (3).

The high contamination and rapid spread capacity of COVID-19 represents a high risk of collapse to health systems, because of the exponential increase demand for healthcare professionals, and semi-intensive and intensive care units for the severe cases (4). The absence of specific preventive or therapeutic medical interventions thus lead the governments to adopt urgent measures to contain the spread of the virus, which included recommendations of social distancing, home confinement, quarantine and/or lockdown. However, despite the effectiveness of these measures for reducing incidence and mortality of COVID-19 (5), they result in negative behaviors that have clinical repercussions for both COVID-19 and global burden of diabetes and others NCDs.

Preliminary findings of an international online survey showed substantial reduction in physical activity levels, increase daily sitting time, and more unhealthy food consumption and meal pattern during COVID-19 home confinement (6). A recent meta-analysis suggested that prolonged TV-viewing time (i.e.: sedentary behavior) was associated with increased risk for type 2 diabetes, cardiovascular disease, and all-cause mortality (7). Sedentary behavior (any waking behavior characterized by an energy expenditure ≤1.5 metabolic equivalents, while in a sitting, reclining or lying posture) has also emerged as a potential risk factor for many chronic conditions and mortality during the last decade (7), and has increased concern during home confinement (8). It is important to note that sedentary behavior is distinct from physical activity levels (9). For example, even with high levels of physical activity, the risk of death associated with high TV-viewing time does not attenuate (7). Furthermore, previous studies have showed that the maintenance of these negative behaviors for few weeks result in deleterious metabolic consequences (impairments in glycemic control, total body fat, abdominal fat, and inflammatory cytokine) that impact management of diabetes and others NCDs (10). In addition, physical inactivity-derived metabolic consequences may have more serious consequences for diabetic individuals during the COVID-19 outbreak, because an adequate glycemic control is associated with a markedly lower mortality rate and disease complications in COVID-19 patients with diabetes (3). Moreover, the potential requirement of cocooning (a more severe form of physical distance measures) or prolonging of home confinement of high risk populations (11), will probably exacerbate the deleterious effects of physical inactivity in individuals with or at risk for diabetes.

However, the recommendation for maintaining adequate levels of physical activity and avoiding sedentary behavior is not always addressed in clinical practice. For example, a recent recommendation for clinical management of diabetes during COVID-19 does not mention the key role of physical activity for maintaining adequate glycemic control and others comorbidities that are highly prevalent in individuals with diabetes (12). Therefore, the present manuscript addresses the consequences of physical inactivity and sedentary behavior during COVID-19 pandemic in individuals with or at risk for diabetes, and the urgent need for recommending physical activity and exercise during and beyond the current outbreak.

## Diabetes, Glycemic Control, and COVID-19 Outcomes

Diabetes is one of the leading NCDs that affects nearly 1 in 11 adults worldwide (9.3% of prevalence) (13). It is strongly associated with disabling and life-threatening health complications (e.g., cardiovascular disease, neuropathy, nephropathy), and a poor management of the disease can result in innumerous and serious complications (13). In this context, nearly 4.2 million adults died from diabetes or its complications (equivalent to one death every 8 s) in 2019 (13). Not surprisingly, diabetes is the second most prevalent NCDs in individuals requiring treatment for COVID-19 and is highly correlated with disease severity (2). A meta-analyses with 24 studies (10,948 patients with COVID-19) found that diabetes was present in 10.0% (95% CI 8.0% to 12.0%) of patients with COVID-19, and that it was strongly correlated with risk of disease severity (OR 2.61, 95% CI 1.93 to 3.52) (2). In agreement, another recent meta-analysis founded that COVID-19 patients previously diagnosed with diabetes have increased risk of severe COVID-19 infection (OR: 2.60, 95% CI: 1.96 to 3.45) and mortality (OR 2.03, 95%CI: 1.29 to 3.20) (14). A more recent multicenter study from a cohort of 7,337 confirmed COVID-19 cases enrolling among 19 hospitals found that individuals with type 2 diabetes required more medical interventions, and had higher mortality (7.8% *versus* 2.7%, adjusted HR = 1.49) and multiple organ injury than non-diabetic individuals (3).

**Figure 1:**
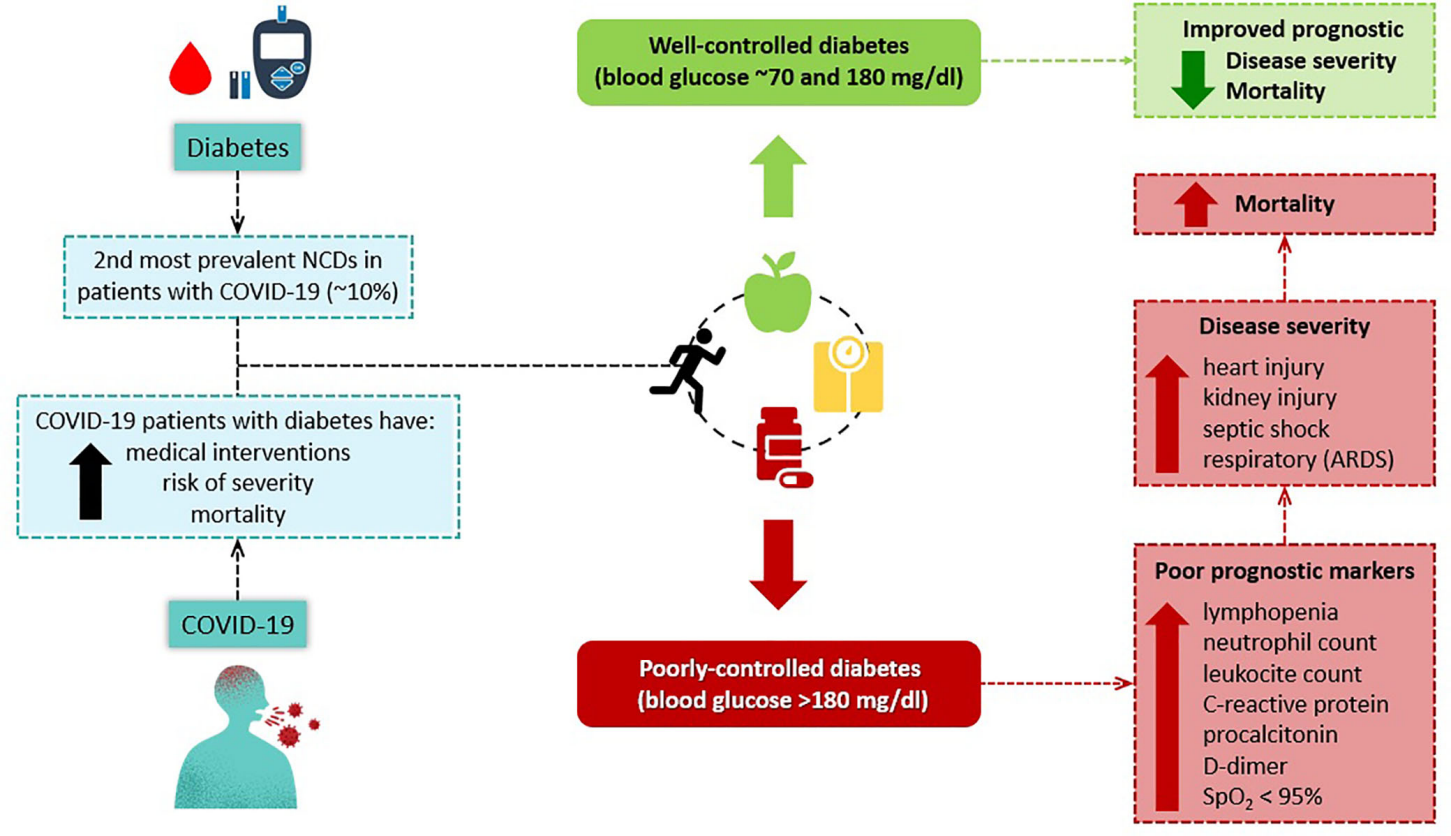
Association between diabetes, glycemic control and COVID-19 severity and mortality.

The current findings are even more alarming when the level of glycemic control is taking into account (**Figure 1**). For example, the above mentioned cohort also compared COVID-19 outcomes between patients with poorly-controlled diabetes (blood glucose >180 mg/dl) and well-controlled diabetes (blood glucose between 70 and 180 mg/dl) and found that patients with poorly-controlled diabetes have significant higher incidence of poor prognostic markers (higher rates of: lymphopenia, 49.6% *vs.* 30.5%; neutrophil count, 19.4% *vs.* 10.7%; leukocyte count, 12.2% *vs.* 6.3%; C-reactive protein, 59.5% *vs.* 47.5%; procalcitonin, 35% *vs.* 24.2%; D-dimer, 55.4% vs. 35.6%; and oxygen saturation of < 95%, 22.7% *vs.* 12.6%), significant increase in severity (Acute Respiratory Distress Syndrome [ARDS], 21.4% *vs.* 7.1%; acute heart injury, 9.9% *vs.* 1.4%; acute kidney injury, 3.8% *vs.* 0.7%; and septic shock, 4.7% *vs.* 0.0%), and higher mortality rate (well-controlled *vs.* poorly-controlled diabetic adjusted HR for all-cause mortality = 0.13, 95% CI 0.04 to 0.44) (3). In addition, the authors also compared outcomes by matching patients 1:1 for other comorbidities (hypertension, cardio- and cerebrovascular disease and chronic kidney disease), and the increase in severity (ARDS, 14.8% *vs.* 7.2%; acute heart injury, 6.8% *vs.* 1.6%; acute kidney injury, 3.2% *vs.* 0.4%), and higher mortality rate (well-controlled *vs.* poorly-controlled diabetic adjusted HR for all-cause mortality = 0.14, 95% CI 0.03 to 0.60) were maintained despite the adjustment (3).

It is important to note that other comorbidities commonly prevalent in individuals with diabetes (i.e.: hypertension, obesity, cardiovascular disease and dyslipidemia) are also associated with high risk of COVID-19 severity and/or mortality (2, 15, 16). For example, the severe acute respiratory syndrome coronavirus 2 (SARS-CoV-2) penetrates human cells through angiotensin-converting enzyme 2 (ACE2), which is more pronounced in adipose tissue and, consequently, in obese population (17). In this context, it is reasonable to suggest that individuals with diabetes should urgently intensify the metabolic control (12), as well as the management of other comorbidities, as a primary prevention of COVID-19.

## Physical Inactivity During COVID-19 Outbreak and Its Impact on Global Burden OF Diabetes and Other NCDs

The absence of specific preventive or therapeutic medical interventions for COVID-19 may require the prolonging of preventive measures (i.e.: social distancing, home confinement and quarantine) by high risk populations, which include individuals with diabetes. In addition, it has been suggested that these high-risk populations should adhere to cocooning (a more severe form of physical distance measures) throughout the COVID-19 outbreak (11). However, although these preventive measures are effective for reducing the incidence and mortality of COVID-19 (5), they may result in negative behaviors that have clinical repercussions for both diabetes and COVID-19 management.

Preliminary results from the ECLB-COVID19 International Online Survey showed substantial reductions in levels of physical activity of all intensities domains (vigorous intensity: ~33.1%, from ~39 to ~26 min/week; moderate intensity: ~33.4%, from ~32 to ~21 min/week; walking: ~34%, from ~37 to ~25 min/week) during COVID-19 home confinement, totaling an average reduction of ~33.5% (from ~108 to ~72 min/week) (6). A ~28.6% (3.10 h) increase in day sitting time (from ~5.31 to ~8.41 h per day), and a more unhealthy food consumption and meal pattern during COVID-19 home confinement (6). Despite of the lack of studies assessing the health impact of these behaviors alterations during the COVID-19 pandemic, it may have important public health implications, which would include an increase of the global burden of diabetes and others NCDs, as well as a poor management of COVID-19 outcomes.

**Figure 2:**
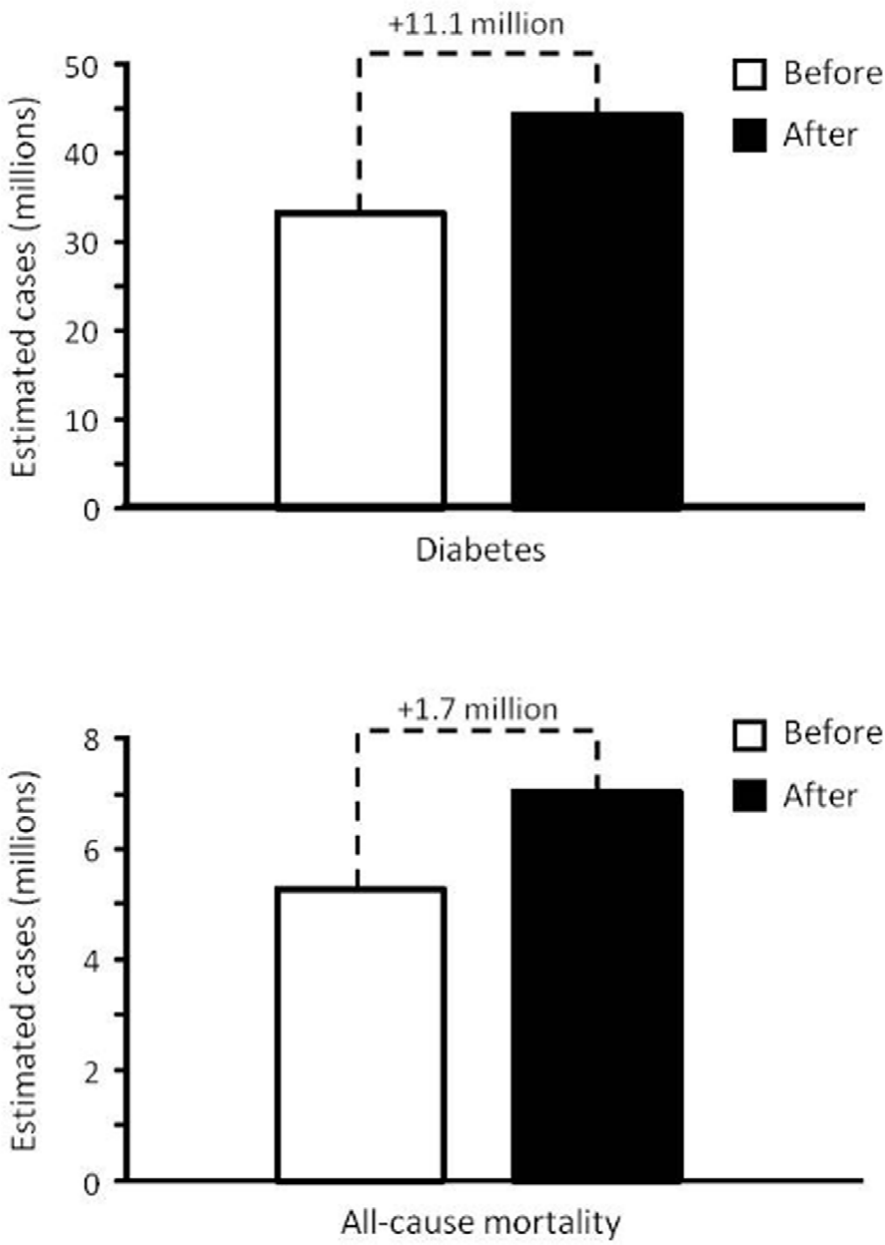
Estimated cases of diabetes and all-cause mortality attributed for physical inactivity, before and after COVID-19 pandemic. Cases were estimated by attribution factors, using the adjusted relative risk of physical inactivity for type 2 diabetes and all-cause mortality (19) and the preliminary findings of physical activity during COVID-19 home confinement (6).

It is estimated that physical inactivity, an activity level insufficient to meet current recommendations (18), is responsible for 7.2% (3.9% to 9.6%) of the cases of type 2 diabetes (~33.3 million cases in 2019) and 9.4% (5.1% to 12.5%) of all-cause mortality (~5.3 million deaths in 2018) worldwide (19). Prior to COVID-19 pandemic, worldwide prevalence of physical inactivity among the population (aged ≥ 40 years) and individuals at risk for type 2 diabetes was estimated to be 42.9% (23.4% to 57.1%) and 43.2% (23.6% to 57.6%), respectively (19). Supposing that the prevalence of physical inactivity during COVID-19 pandemic increased at the same rate as total level of physical activity (~33.5%) (6), the prevalence of physical inactivity is currently 57.3% (31.2% to 76.2%) and 57.7% (31.5% to 76.9%) among the population (aged ≥ 40 years) and individuals at risk for type 2 diabetes, respectively. In this context, by using population attribution factors and the known adjusted relative risk of physical inactivity for type 2 diabetes and all-cause mortality (19), we can estimate that physical inactivity will be responsible for 9.6% (5.3% to 12.8%) of the cases of diabetes and 12.5% (6.8% to 16.7%) of all-cause mortality worldwide during the COVID-19 pandemic. Thus, the cases of type 2 diabetes and all-cause deaths attributed to physical activity would increase by ~11.1 and ~1.7 million during COVID-19 pandemic, respectively (**Figure 2**). Noteworthy, a recent study showed that physical inactive is associated with a greater relative risk for hospitalization for COVID-19, even after adjustment for age, sex, obesity, smoking and alcohol consumption (relative risk 1.32, 95% CI 1.10 to 1.58) (15), suggesting that an increase in the prevalence of physical inactivity may also result in the increase of COVID-19 hospitalizations.

Previous studies have also showed that the maintenance of negative behaviors (i.e.: physical inactivity, sedentary behavior, and unhealthy food consumption) for few weeks result in deleterious effects on metabolic (increases in insulin resistance, total body fat, abdominal fat and inflammatory cytokines), immune function (immunosenescence), and cardiovascular parameters (blood pressure and heart rate increase, endothelial function reduction, etc…) that impact the management of diabetes and others NCDs (10, 20–22). For example, substantial worsening of glycemic control and reduced rate of muscle protein synthesis has occurred in overweight and pre-diabetic older individuals who reduced the daily walking to less than 1,000 steps per day for two weeks (23), which may be easily meet during home confinement. In addition, the impairments in glycemic control and rate of muscle protein synthesis were still present after 2 weeks of resuming to baseline daily walking levels (23).

**Figure 3:**
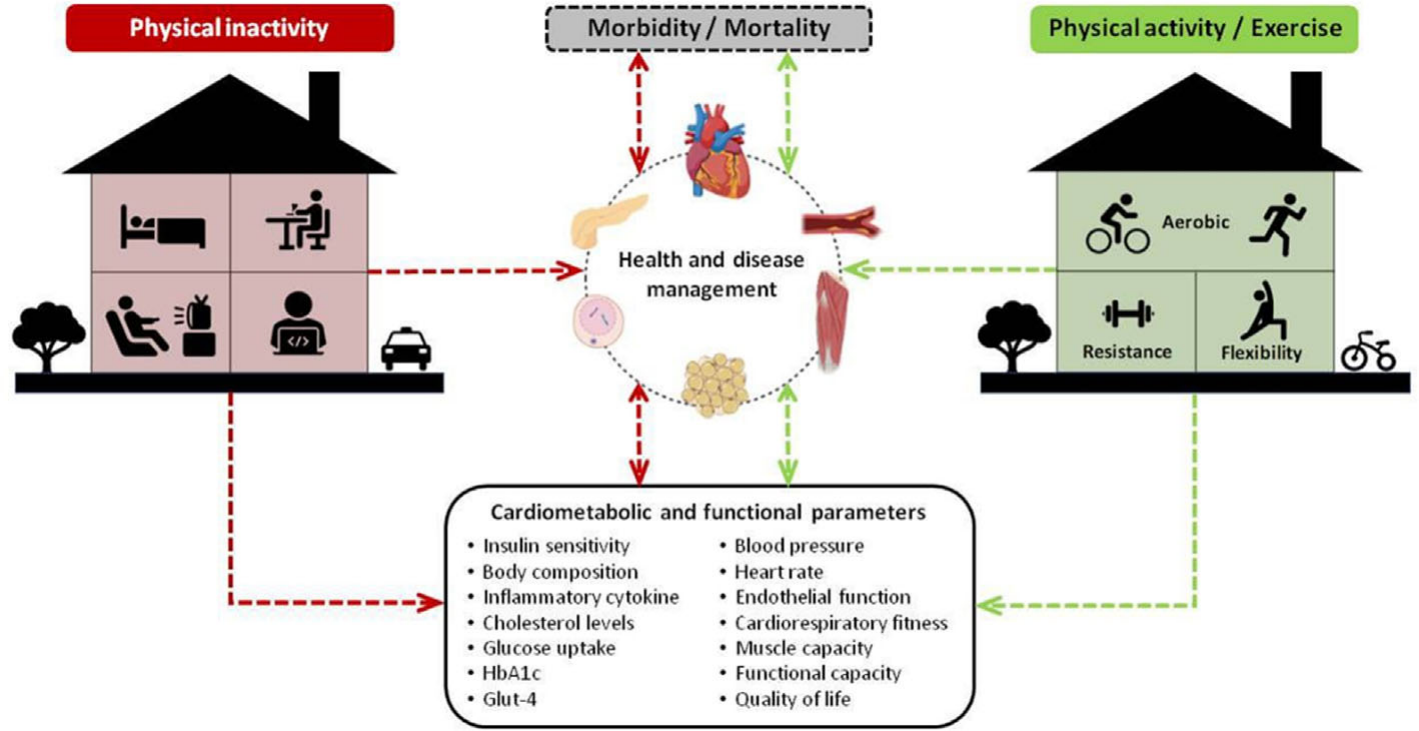
Consequences of physical inactivity (negative effects) and physical active/exercise (positive effects) during home confinement on cardiometabolic and functional parameters, health and disease management, and morbidity/mortality.

The reduction of physical activity during home confinement may also have health consequences to individuals with diabetes that are previously active. Studies assessing the effects of exercise detraining in individuals with diabetes that were previously performing regular exercise programs showed innumerous physiological (i.e.: increase in resting heart rate and body fat, dysregulation of insulin and glucose secretion, decrease in the levels of GLUT-4 transporter, loss of training-induced improvements in cholesterol and HbA1C levels) and functional consequences (i.e.: reduction of aerobic, muscle strength, flexibility, balance and agility performance) after short periods of interruption of aerobic and/or resistance exercise programs (24–29). It is important to note that the potential requirement of cocooning (a more severe form of physical distance measures) or prolonging of home confinement of high risk populations (11) will probably exacerbate the above mentioned deleterious effects of physical inactivity, and contribute to negative psychological effects (i.e.: quarantine duration, infection fears, frustration, boredom, inadequate supplies) (30) in individuals with or at risk for diabetes. In this context, the maintenance or increase of physical activity levels, as well as the avoidance of sedentary behavior (**Table 1**), should be emphasized during and beyond COVID-19 pandemic to prevent the severity of COVID-19, as well as to prevent the deleterious effects of physical inactivity on the management of diabetes and others NCDs (**Figure 3**), which may positively impact others syndemics (i.e.: food insecurity, malnutrition and obesity) (31), epidemics (i.e.: obesity) (17) and pandemics (i.e.: sedentary behavior) (32).

**Table 1:** Reducing sedentary behavior by WHO guidelines.

	Activity	Description	Action
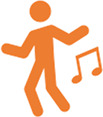	Take short active breaks during the day	Short bouts of physical activity add up to the weekly recommendations;	Dancing, playing with children, and performing domestic chores (i.e.: cleaning and gardening) and other means to stay active at home.
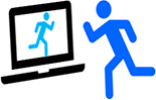	Follow an online exercise class	Take advantage of the wealth of online exercise classes;	Many of these are free and can be found on YouTube. If you have no experience performing these exercises, be cautious and aware of your own limitations.
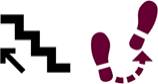	Walk	Walking around or walking on the spot, can help you remain active;	If you have a call, stand, or walk around your home while you speak, instead of sitting down.
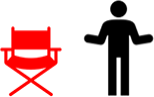	Stand up	Interrupt sitting and reclining time every 30 min;	Reduce your sedentary time by standing up whenever possible. During sedentary leisure time prioritize cognitively stimulating activities (i.e.: reading, board games, and puzzles).
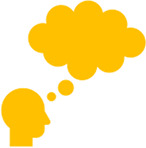	Relax	Meditation and deep breaths can help you remain calm;	Sit comfortably or legs up the wall. Concentrate on your breath, trying not to focus on any thoughts or concerns. Stay comfortable, relaxing and de-stressing.
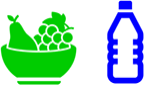	Eat healthily and stay hydrated	Ensure plenty of fruits and vegetables, and limit the intake of salt, sugar and fat. Limit or avoid alcoholic drinks;	Plan your intake. Use fresh ingredients. Be aware of portion sizes. Avoid drinking caffeinated and energy drinks. Drinking water instead of sugar-sweetened beverages.

Available at: https://www.euro.who.int/en/health-topics/health-emergencies/coronavirus-covid-19/technical-guidance/stay-physically-active-during-self-quarantine.

## Physical Exercise for Managing Diabetes and Counteracting Deleterious Effects of Home Confinement During COVID-19 Outbreak

The benefits of behavioral interventions for promoting metabolic and cardiovascular benefits are well established (33, 34), with the probability that behavioral interventions are 59% more effective than pharmaceutical treatments for reversing of metabolic syndrome components (20, 21). Regarding to prevention and treatment of diabetes, lifestyle interventions are recommended to be based on a well-structured physical activity program (physical exercise) and a healthy nutritional behavior (13), and physical exercise is essential for improving glycemic control, insulin signaling, blood lipids, low-grade inflammation, vascular function, body composition and others health variables (35).

Systematic reviews with meta-analysis have showed that most of above mentioned benefits of physical exercise can be obtained by aerobic and/or resistance exercise programs (25, 36–39). For example, a recent-meta-analysis of 37 studies involving 2208 individuals with diabetes showed that both supervised aerobic or resistance exercise were effective for promoting substantial improvements in HbA1C, total cholesterol and triglycerides; however, only aerobic exercise improved fasting plasma glucose and low-density lipoprotein cholesterol, while only resistance exercise improved systolic blood pressure (37). It is important to note that the benefits of aerobic exercise appears to be associated with its intensity, with high-intensity exercise inducing superior effects in HbA1c, insulin, body weight, body mass index, VO_2MAX_, lipid profile, C reactive protein, interleukin 6, and systolic blood pressure when compared to low and moderate intensities (25, 36, 38).

In this context, in order to maintain or improve the health condition of individuals with or at risk for diabetes, current guidelines recommends at least 150 min (30 min, 5 d/wk) of moderate-intensity exercise (40-60% VO_2MAX_) or 75 min (25 min, 3 d/wk) of high-intensity exercise (60-85% of VO_2MAX_) per week, in association with 2 to 3 sessions per week of resistance exercise (40–43). Flexibility and balance training (i.e.: yoga, tai chi) are also recommended (2-3 d/wk) mainly for older individuals (43). In addition, in order to decrease the daily sedentary behavior, it is also recommended to interrupt prolonged sitting every 30 min (43). Indeed, according with recent findings, the replacement of sedentary behavior with light intensity physical activity (100 to 1951 counts/min) might be beneficial for diabetes risk markers (44). As 10.5 min of light intensity physical activity is equivalent to 1 min of moderate-to-vigorous physical activity, to performing higher volumes of light intensity physical activity is a beneficial alternative to improve cardiometabolic health in individuals who cannot meet the guidelines recommendations due to any reason (45). Finally, these and others recommendations also emphasize the importance of combining aerobic and resistance exercise to optimize improvements on glucose control, HbA1c, blood lipids, body composition, systolic blood pressure and liver and pancreatic function (37, 40, 41, 46, 47).

Despite of the frequency, intensity, time and type (FITT) of exercise recommended for individuals with or at risk for diabetes (40, 41) (**Table 2**), lower frequencies and/or time (duration) of exercise are effective. For example, high-intensity exercise programs with a weekly time commitment 25% to 56% lower than the minimum recommended in current exercise guidelines showed significant improvements in blood glucose, HbA1c, lipid profile (i.e.: total cholesterol, high-density lipoprotein and triglycerides), blood pressure, endurance performance, body composition in individuals type 2 diabetes mellitus (48) or with overweight/obesity and dyslipidemia (49). Only one session of exercise is effective to transiently reduce capillary glycemia, insulin sensitivity, and ambulatory blood pressure in individuals with diabetes (35, 50). In addition, the breaking up of sedentary behavior with very-short bouts of moderate- or high-intensity exercise throughout the day (“exercise snacks”) has shown several health-related benefits, including improvements in cardiorespiratory fitness (51), vascular function (52), glycemic control (53) and muscle function (54), being suggested as an effective strategy to prevent some deleterious effects of sedentary behavior and unhealthy food consumption (52). Indeed, the benefits of exercise snacks were reached by simple exercises using body weight (e.g.: stair climbing, sit-to-stand from a chair, marching on the spot…). Other options without specific materials include the use of items with light and moderate weights (e.g.: rice bags, battle of water), walking inside the house, dancing or balance exercise, and stepping over obstacles (30). In this context, low frequencies and/or time (duration) of exercise have several health-related benefits and should be encourage for those individuals unable to meet the minimum FITT recommendation.

**Table 2:** Minimal exercise frequency, intensity, time, and type recommendations for individuals at risk or with type 2 diabetes.

Modality	Frequency	Intensity	Time	Type
Aerobic	At least 3 d/wk	Moderate: 40% to 59% of HR reserve, 12 to 13 in the 6-20 RPE, 3 to 4 in the CR-10 or comfortable conversation possible (Talk Test)Vigorous: 60% to 89% of HR reserve, 14 to 17 in the 6-20 RPE, 5 to 7 in the CR-10 or comfortable conversation not likely possible (Talk Test)	25 to 60 min/d (at least 150 min/wk of moderate intensity or 75 min/wk of vigorous intensity) in bouts of 10 min or more	Continuous activities using major muscle groups (e.g.: walking, jogging, running, cycling, dancing, climbing stairs, jumping jacks, skipping rope…)
Resistance	At least 2-3 d/wk	Moderate to vigorous intensity: 60%-80% 1-RM, 14 to 17 in the 6-20 RPE or 5 to 7 in the CR-10	No specific duration. 1 to 4 sets of 8 to 15 reps, with 1 to 2 min of interval between sets, and performed in 6 to 10 exercises (1 exercise for each major muscle groups)	Resistance based-activities (e.g.: weight lifting exercises, body-weighted exercises [squats, push-ups, sit-ups, abdominal crunch]…)
Balance*	2-3 d/wk	No specific intensity	No specific duration	Activities that progressively reduce the base of support, perturb the center of gravity, stress postural muscle groups, and/or reduce sensory input (e.g.: tai chi chuan, two-legged stand, semitandem stand, tandem stand, one-legged stand, tandem walk, circle turns, heel stands, toe stands, standing with eyes closed…)
Flexibility*	2-3 d/wk	Moderate: 13 to 15 in the 6-20 RPE scale or 5 to 6 in the CR-10	No specific duration. All major muscle groups should be stressed	Activities that maintain or increase flexibility (e.g.: yoga, sustained stretches)

6-20 RPE: 6 to 20 rating of perceived exertion scale; CR-10: category ratio scale. *Balance and flexibility are recommended only for older individuals (age > 65 years).

As part of the necessary social distancing measures during COVID-19 outbreak, the use of public spaces, athletic clubs, gyms and health centers for practicing exercise is not recommended (or permitted), mainly for high-risk populations. Thus, home-based exercise training emerges as the most important potential approach to control, maintain or increase the exercise practice during the COVID-19 pandemic. Further, home-based training has several potential advantages (i.e.: expanded access, individual programs, flexible scheduling, individuals’ privacy and, an integration with regular home routine) (55), and has been safe and effective for individuals with diabetes (**Table 3**). Randomized controlled trials assessing the benefits of home-based exercise programs in individuals with diabetes showed positive effects on glycemic control (56–60), lipid profile (56), body composition (61–63), cardiorespiratory fitness (i.e. exercise capacity, maximal oxygen uptake) (62–65) and psychological variables (62, 65). In addition, the adherence to a home-based exercise program was strongly associated with a reduced incidence of CVD among individuals with type 2 diabetes (a 10-fold higher risk among individuals who dropped out when compared with individuals who completed the home-based exercise program) (66).

**Table 3:** Overview of studies assessing the effects of home-based exercise programs in individuals with diabetes.

Study/population	Home-based and comparator groups (N/age)/Follow-up	Orientation, monitoring and follow-up	Tools and measurements during home-based intervention	Home-based exercise programs	Home-based exercise improvements
Collins et al. (53) /T2DM+PAD	Home-based: 37/35 (M/F)/66 ± 10 yr Control: 53/20 (M/F)/67 ± 10 yr Follow-up: 6 months	Orientation: 7-min educational video/orientation on self-management behaviors/instructional audiotape Monitoring: phone calls (biweekly for 6 months)	Tools: pedometers and questionnaire Measurements: diary (daily glucose, lipid, weekly blood pressures)	Frequency: 4-5 d/wk Intensity: not reported Time: 50 min/session Type: aerobic (walking)	Walking speed; and quality of life
Dadgostar et al. (41) /T2DM	Home-based: 36 (F)/49 ± 6 yr Supervised exercise: 38 (F)/50± 5 yr Follow-up: 3 months	Orientation: general information on diabetes, self-care, diet, and exercise (90min) + educational booklet Monitoring: clinical visit (baseline, week 6 and week 12) + phone calls (biweekly for 6 weeks)	Tools: pedometers, elastic bands and activity log Measurements: not reported	Frequency: 3-5 d/wk Intensity: moderate (gradual progress from 2,500-30,00 to 10,000–12,000 steps per day) Time: not reported Type: aerobic (walking) and resistance (elastic bands)	Glycemic control; body composition; lipid profile; and health-related quality of life
Guelfi et al. (50) /Gestational DM	Home-based: 85 (F)/34 ± 4 yr Control: 87 (F)/34 ± 4 yr Follow-up: 3 months	Orientation: not reported Monitoring: supervision by an exercise physiologist at participants’ home (3 times-a-week)	Tools: HR monitor and RPE scale Measurements: diary (daily nutritional intake)	Frequency: 3 d/week Intensity: moderate (65-75% HR_MAX_) with intervals of high (75-85% HR_MAX_) Time: 20-60 min/session (progressive) Type: aerobic (cycle ergometer)	Cardiorespiratory fitness; exercise automaticity; and general psychological distress
Halse et al. (43) Gestational DM	Home-based: 20 (F)/29 ± 1 yr Control: 20 (F)/29 ± 1 yr Follow-up: 8 months	Orientation: counseling by a diabetes educator and dietician Monitoring: home visit	Tools: exercise diary and RPE scale Measurements: capillary glucose, food diary and questionnaires	Frequency: 5 d/wk Intensity: progressive - moderate- (65-75% HR_MAX_) to high-intensity interval (75-85% HR_MAX_) Time: 25 to 45 min/session (progressive) Type: aerobic (cycle ergometer)	Postprandial glycemic control; and post-exercise capillary glucose
Karjalainen et al. (55) /T2DM+CAD	Home-based T2DM+CAD: 32/7 (M/F)/62 ± 5 yr Home-based CAD:???? (32/12 (M/F)/62 ± 5 yr Follow-up: 6 months	Orientation: not reported Monitoring: contacted by a sports medicine specialist or physiotherapist (1 and 3 months)	Tools: accelerometer, HR monitor and exercise diary Measurements: daily diary	Frequency: 5 d/wk Intensity: 50-70% HR_RESERVE_ (progressive) Time: 60 min/session Type: aerobic and resistance training	Cardiorespiratory fitness; and daily levels of high-intensity activity
Krousel-Wood et al. (46) /T2DM	Home-based: 37 (not reported)/57 ± 10 yr Control: 39 (not reported)/57 ± 10 yr Follow-up: 3 months	Orientation: education program on diabetes self-management (5 sessions, 2.5h) Monitoring: clinic visit (1 per month up to 3^rd^ month)	Tools: videotape exercise and activity logs Measurements: questionnaires	Frequency: 5 d/w Intensity: low- to moderate-intensity (3-6 METs) Time: 30 min/session Type: aerobic and resistance	Body mass index; and quality of life
Lee et al. (44) /T2DM	Home-based steps group: 19/21 (M/F)/54 ± 10 yr Home-based aerobic group: 21/19 (M/F)/56 ± 8 yr Control: 18/22 (M/F)/56 ± 9 yr Follow-up: 3 months	Orientation: a nurse-oriented session on how to correctly perform the program Monitoring: phone calls (weekly)	Tools: pedometer (steps group) or portable oximeter and RPE scale (aerobic group) Measurements: not reported	Frequency: 5 d/wk Intensity: moderate (13-15 RPE) or not reported (steps group) Time: 10,000 steps/day (steps group) or 30 min/session (aerobic group) Type: aerobic (steps group: walking; aerobic group: brisk walking, jogging and/or bicycling)	Glucose metabolism; and pancreatic beta cell function (greater improvements in the steps group)
Marios et al. (49)/T2DM	Tele-monitored home-based: 10/5 (M/F)/60 ± 9 yr Non-monitored home-based (control): 4/9 (M/F)/65 ± 8 yr Follow-up: 6 months	Orientation: not reported Monitoring: phone calls (weekly)	Tools: HR monitor Measurements: exercise training diary	Frequency: not reported Intensity: not reported Time: 180 min per week Type: aerobic (walking program)	Cardiorespiratory fitness; and exercise tolerance
Olse et al. (45)/T2DM	Home-based T2DM: 9 (M)/60± 2 yr Home-based healthy control: 8 (M)/56 ± 1 yr Follow-up: 2 months	Orientation: not reported Orientation: regular phone calls	Tools: HR monitor Measurements: exercise training diary	Frequency: 3-4 d/wk Intensity: 65-70% VO_2PEAK_ Time: 30 min/session Type: aerobic (rowing ergometer)	Submaximal aerobic capacity; and insulin‐mediated glucose extraction and clearance
Plotnikoff et al. (42) /T2DM	Home-based: 8/19 (M/F)/55 ± 12 yr Control: 8/13 (M/F)/54 ± 12 yr Follow-up: 4 months	Orientation: one week of learning and practicing of each exercise by supervision of an exercise specialist Monitoring: home visits (18 of 48 sessions) + clinical visits (week 2 and 10)	Tools: multigym apparatus and dumbbells Measurements: exercise training logs	Frequency: 3 d/week Intensity: moderate- (50-60% of 1RM) to high-intensity (70-85% of 1RM) - progressive Time: not reported (2-3 sets of 8-12 reps in 8 exercises) Type: resistance	Muscle strength; fasting insulin; HDL cholesterol; social-cognitive variables; and exercise self-efficacy
Scheede-Bergdahl et al. (52) /T2DM	Home-based T2DM: 12 (M)/59 ± 2 yr Home-based healthy control: 9 (M)/55 ± 1 yr Follow-up: 2 months	Orientation: not reported Monitoring: not reported	Tools: HR monitor Measurements: training logs	Frequency: 3-4 d/wk Intensity: 65-70% of VO_2PEAK_ Time: 30 min/session Type: aerobic (rowing ergometer)	Submaximal aerobic capacity; and C-reactive protein
Shinji et al. (51) /T2DM	Home-based high-compliance: 40/24 (M/F) 58 ± 10 yr Home-based low-compliance: 21/17 (M/F)/54 ± 10 yr Follow-up: 3/17 months of intervention/incidence of cardiovascular events	Orientation: diabetes education, health counseling and an exercise prescription Monitoring: phone calls	Tools: not reported Measurements: self-reported adherence	Frequency: 4-6 d/wk Intensity: moderate (adjusted to anaerobic threshold) Time: 20-30 min/session Type: aerobic (walking)	Lower incidence of cardiovascular disease
Wu et al. (47) /at risk for T2DM	Home-based: 22/46 (M/F)/54 ± 5 yr Control: 16/51 (M/F)/54 ± 6 yr Follow-up: 9 months	Orientation: educational orientation with a physiotherapist (1.5h), and guided book on proper diet and diabetes prevention Monitoring: phone calls (weekly–biweekly intervals for 3 months, reducing from 3 to 6 months and ending after 6 months)	Tools: exercise video and stepper Measurements: body weight, exercise training logs and questionnaires (physical activity, self-efficacy)	Frequency: 3-5 d/wk Intensity: moderate to vigorous Time: 30 min/session Type: aerobic	Exercise self-efficacy; body mass index; muscle endurance; flexibility; and physical activity levels
Yang et al. (48) /T2DM	Home-based: 274/309 (M/F)/58 ± 1 yr Follow-up: 6 months	Orientation: education on diabetes management and healthy lifestyle behaviors Monitoring: supervised session once-a-week	Tools: not reported Measurements: exercise training diary	Frequency: 5 d/wk Intensity: 60-75% of VO_2PEAK_ or HR_RESERVE_ Time: not reported Type: aerobic (walking) and resistance (free weights/elastic bands)	Cardiorespiratory fitness; and body mass index

CAD, coronary artery disease; DM, diabetes mellitus; F, female; HR, heart rate; M, male; PAD, peripheral arterial disease; RPE, rating of perceived exertion; T2DM, type 2 diabetes mellitus.

Moderate-intensity continuous aerobic exercise (3 to 5 times per week), regulated by VO_2PEAK_/HR_RESERVE_ (58, 60, 63, 65), METs (61) or steps (59), was the most frequent modality in the studies involving home-based training (**Table 2**); however, programs with combined aerobic and resistance exercise (56, 61) or multicomponent exercise (57) were also used. Some studies provided equipment’s to control the exercise performed at home, such as heart rate monitor (60, 64), pedometers (56, 59, 61), potable oximeter (59), cycle and home rowing ergometer (65, 67). However, these tools may be expensive, difficult to administer and not suitable for all participants in unsupervised sessions. In this sense, RPE scale, talking test or questionnaires can be a feasible and cost-effectiveness alternative in controlling the sessions (58, 59, 65). In addition, the use of personal glucometers, food intake diary and body weight variables are also alternatives to control the responses of exercise sessions in physiological variables (58, 62, 65). It is important to emphasize that there were no adverse events reported during the follow-up of all home-based exercise program (57–60, 62–69), which can be explained by the fact that most studies provided a first orientation session involving explanations about exercise program and/or a complex diabetes self-management education (56–59, 61–63, 65, 70). Finally, home-based exercise training appears to be more cost-effective than traditional exercise programs performed in centers (71). In this context, despite the absence of social interaction, as well as the lack of studies assessing the effectiveness in individuals with type 1 diabetes or the safety of high-intensity exercise, home-based exercise programs are useful, safe and effective for the management of diabetes, especially during COVID-19 outbreak.

## Conclusion

Despite of the lack of studies assessing the health impact of the negative behaviors during the COVID-19 pandemic, physical inactivity may have important public health implications, including an increase in global burden of diabetes and other NCDs, as well as impaired COVID-19 management. These deleterious effects of physical inactivity can be exacerbated by the potential requirement of cocooning or prolonging of home confinement of high-risk populations. In contrast, physical activity and exercise are important tools for preventing and treating diabetes and others NCDs. In addition, home-based exercise programs are useful, safe, and effective for the management of diabetes, and could be widely used during COVID-19 outbreak. In this context, there is an urgent need for recommending physical activity/exercise, during and beyond COVID-19 outbreak, for improving the management of diabetes, as well as to prevent the increase in global burden of COVID-19, diabetes and others NCDs.

## Authors’ Contributions

IRM, BF, AAV, and EGC conceived, designed, and drafted the manuscript; IRM and EGC prepared figures and tables. IRM and EGC edited and revised the manuscript. All authors contributed to the article and approved the submitted version.

## Funding

IRM, BF, and EGC were supported by Fundação de Amparo à Pesquisa do Estado de São Paulo (FAPESP #2018/09695-5–FAPESP #2019/19596-7), Comissão de Aperfeiçoamento de Pessoal de Nível Superior (CAPES—Finance Code 001), and Conselho Nacional de Desenvolvimento Científico e Tecnológico (CNPq #303399/2018-0), respectively.

## Conflict of Interest

The authors declare that the research was conducted in the absence of any commercial or financial relationships that could be construed as a potential conflict of interest.
